# Transorbital Sonographic Evaluation of Normal Optic Nerve Sheath Diameter in Healthy Volunteers in Bangladesh

**DOI:** 10.1371/journal.pone.0081013

**Published:** 2013-12-02

**Authors:** Rapeephan R. Maude, Md Amir Hossain, Mahtab Uddin Hassan, Sophie Osbourne, Katherine Langan Abu Sayeed, Mohammed Rezaul Karim, Rasheda Samad, Shyamanga Borooah, Bal Dhillon, Nicholas P. J. Day, Arjen M. Dondorp, Richard J. Maude

**Affiliations:** 1 Mahidol-Oxford Tropical Medicine Research Unit, Rajthewi, Bangkok, Thailand; 2 Centre for Tropical Medicine, Nuffield Department of Medicine, University of Oxford, Oxford, United Kingdom; 3 Chittagong Medical College Hospital, Chittagong, Bangladesh; 4 College of Medicine and Veterinary Medicine, University of Edinburgh, Edinburgh, United Kingdom; Charité University Medicine Berlin, Germany

## Abstract

**Introduction:**

Measurement of optic nerve sheath diameter (ONSD) by ultrasound is increasingly used as a marker to detect raised intracranial pressure (ICP). ONSD varies with age and there is no clear consensus between studies for an upper limit of normal. Knowledge of normal ONSD in a healthy population is essential to interpret this measurement.

**Methods:**

In a prospective observational study, ONSD was measured using a 15 MHz ultrasound probe in healthy volunteers in Chittagong, Bangladesh. The aims were to determine the normal range of ONSD in healthy Bangladeshi adults and children, compare measurements in males and females, horizontal and vertical beam orientations and left and right eyes in the same individual and to determine whether ONSD varies with head circumference independent of age.

**Results:**

136 subjects were enrolled, 12.5% of whom were age 16 or under. Median ONSD was 4.41 mm with 95% of subjects in the range 4.25–4.75 mm. ONSD was bimodally distributed. There was no relationship between ONSD and age (≥4 years), gender, head circumference, and no difference in left vs right eye or horizontal vs vertical beam.

**Conclusions:**

Ultrasonographic ONSD in Bangladeshi healthy volunteers has a narrow bimodal distribution independent of age (≥4 years), gender and head circumference. ONSD >4.75 mm in this population should be considered abnormal.

## Introduction

Identification of elevated intracranial pressure (ICP) is important in the assessment of a range of neurological diseases. It is a predictor of poor prognosis including risk of death from brainstem herniation. ICP is commonly measured by opening pressure on lumbar puncture but this is invasive, unpleasant for the patient, and contraindicated in many cases. Non-invasive detection of raised ICP can be achieved by detection of specific signs e.g. papilloedema on fundoscopy. This requires an experienced examiner with an ophthalmoscope and the changes can appear late requiring a sustained increase in ICP.[1], [2] Computed tomography (CT) and magnetic resonance imaging (MRI) of the head can be used to infer ICP and determine the safety or otherwise of lumbar puncture. These require the patient to be moved, are frequently not available in resource-poor settings and can be normal early in the presence of raised ICP.[3], [4]_ENREF_2

Ultrasound measurement of the optic nerve sheath diameter (ONSD) is a quick, non-invasive method of detecting raised ICP. It is increasingly being used in emergency departments and intensive care units.[5], [6], [7] The optic nerve sheath is a membrane covering the optic nerve behind the eye and is continuous with the dura mater over the brain. It distends when ICP is high due to expansion of the underlying subarachnoid space and its' diameter can be reliably measured at its point of maximal distension 3 mm posterior to the globe.

ONSD has been used as a clinical and research tool for a variety of conditions to detect raised ICP. Its sensitivity for detecting raised ICP is high[5], [8] and ONSD varies almost concurrently with ICP.[9]_ENREF_7 It is cheap and easy to train operators. Ultrasound is known to be operator dependent. However, ONSD ultrasound has been well evaluated and found to have low intra and inter-operator variability.[10], [11]_ENREF_9 ONSD can be measured with the ultrasound beam in the vertical or horizontal orientation and this varies between studies. The degree of normal intra-individual variation in ONSD between left and right eyes is thought to be minimal.[2],[3],[12]

There is no consensus as to the cut-off for an abnormal ONSD indicating raised intracranial pressure. There is considerable interindividual variation[9] but no difference between male and female children.[12] Although 5 mm is most commonly used for adults, different studies have used values up to 5.9 mm.[9] In children, ONSD has been shown to increase with age with most of the increase in the first year of life. Threshold values of 4.0 mm under 1 year and 4.5 mm in those 1 to 16 years of age [9], [12] or 4 mm under 4 years and 5 mm in older children and adults[2] have been proposed._ENREF_8 It is not known whether ONSD increases beyond childhood[12] or how ONSD varies with ethnicity or head circumference.

Knowledge of the normal range of ONSD in a healthy population is essential to interpret this measurement as a marker of intracranial pressure in clinical practice[5], [13] and research. Studies are currently underway in Bangladesh using ONSD to detect raised intracranial pressure in adult severe malaria. To date, however, there has been no evaluation of ONSD in a healthy Bangladeshi population.

An observational study was performed to determine the normal range of ONSD in healthy Bangladeshi adults and children, compare measurements in males and females, horizontal and vertical beam orientations and left and right eyes in the same individual and to determine whether ONSD correlates with head circumference independent of age.

## Materials and Methods

The study was conducted in Chittagong Medical College Hospital, Chittagong, Bangladesh. Chittagong Medical College Ethical Committee and OXTREC, the University of Oxford Tropical Research Ethics Committee (OXTREC reference 24–12) provided ethical approval for this study. Healthy relatives of patients and hospital staff of all ages and both genders were recruited if they provided written, informed consent. Written informed consent was obtained from the next of kin, caretakers, or guardians on behalf of children participating in the study. Individuals were excluded if they had any chronic diseases, any acute illnesses in the preceding 4 weeks or had taken any medications in the preceding 4 weeks. Upon enrollment, basic demographic data were collected and the head circumference measured by a single observer using a purpose-designed non-stretchable tape around the widest possible occipitofrontal circumference.

ONSD was measured 3 mm behind the retina. A single investigator used a 15 MHz linear ultrasound probe (Accutome B-Scan Plus, Accutome Inc., USA) oriented perpendicularly in the vertical plane and at around 30 degrees in the horizontal plane on the closed eyelids of both eyes of supine subjects. Ultrasound gel was applied to the outside of each eyelid and recordings made in the axial and longitudinal planes of the widest diameter visible. Video of every ultrasound was recorded for later analysis by a single blinded investigator. To determine ONSD, electronic calipers were used to mark 3 mm perpendicularly behind the retina. The ONSD was measured at the depth marker at right angles to the optic nerve. This method has been described and illustrated diagrammatically in detail elsewhere.[14] Each video was played three times and a single measurement made each time from a randomly selected frame, giving 6 measurements of each eye and 12 measurements in total per subject.

The study aimed to include 100 healthy Bangladeshi people. Previous studies in healthy children found ranges of ONSD of 2.5–4.1 mm in 31 people in Africa[13] and 2.1–4.3 mm in 102 people in the UK with correlation with age.[12] As it was not known to what degree ONSD varies in Bangladeshi people, or how this changes with age or skull size, it was not possible to perform a precise sample size calculation. The UK study was used as a guide to the approximate sample size required. With this sample size, assuming an alpha of 0.05, two groups of equal size and a total population of 158,000,000, a 10% difference in mean ONSD between adults and children can be detected. Results from a second smaller group of different healthy individuals recruited concurrently from the same population for a separate study were also included for comparison. In this group, videos were recorded by a different investigator from the first group using the same methodology except head circumference was not measured and ONSD was recorded only in the horizontal plane. Results of this study will be published separately. The same investigator measured all ONSDs from the videos for both groups.

Statistical analysis was performed using GraphPad Prism 6 (GraphPad Software, Inc., USA). Mann Whitney U test was used to compare unpaired ONSD between genders and observers, Wilcoxon matched pairs signed rank test to compare left and right eyes and horizontal and vertical measurements. Linear regression was used to assess for correlations with age and head circumference. One way analysis of variance and intraclass correlation coefficient (CCI) were used to test for differences between the repeated measures of ONSD within individuals. Mean values of repeated measures were used for correlations and comparison between groups.

## Results

**Figure 1:**
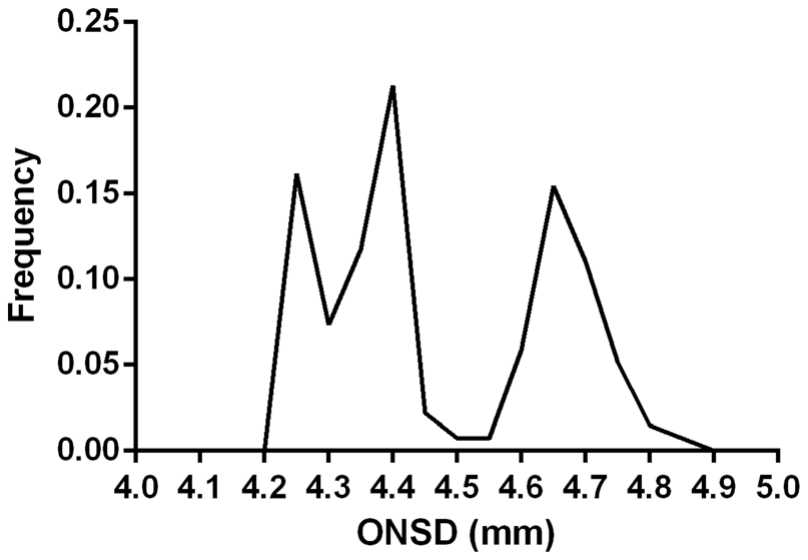
Distribution of mean optic nerve sheath diameter (ONSD) measurements.

ONSD was measured in 106 healthy volunteers by the first observer, and an additional 30 by the second observer. All those asked agree to participate in the study. 17/136 (12.5%) subjects were age 16 or under and 49.3% were male. All patients were of Bangladeshi origin. The median (range) ONSD was 4.41 (4.24–4.83) mm and 95% of individuals had mean ONSD in the range 4.25–4.75 mm (**figure 1**). The distribution of ONSD in this study was bimodal (**figure 1**). There was no difference in the measurements in the two groups [median (range) 4.41 (4.24–4.83) vs 4.33 (4.24–4.75) mm, p = 0.52]. There was no difference between the 6 repeated measures of ONSD in each eye (right eye CCI = 0.897, F (4.432, 465.3)  =  1.331, p = 0.254 and left eye CCI = 0.897, F (4.595, 482.4) = 1.351, p = 0.2451).

**Figure 2:**
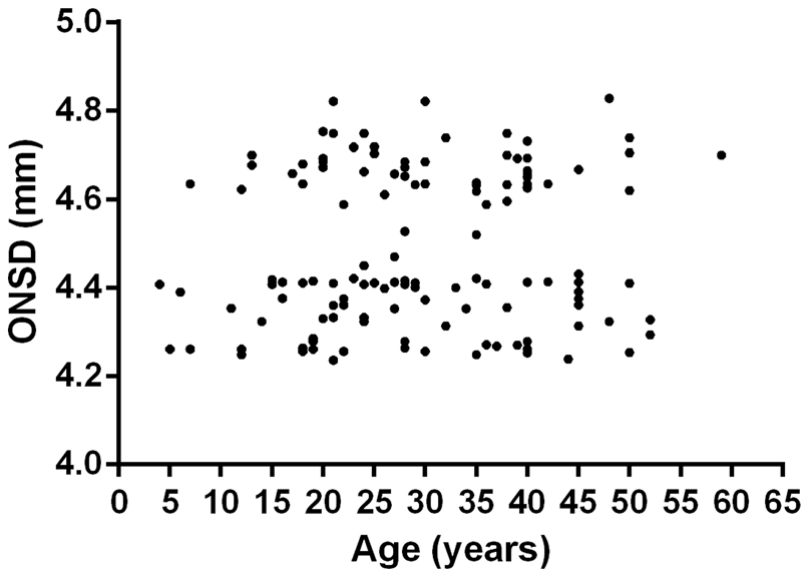
Optic nerve sheath diameter (ONSD) and age.

**Figure 3:**
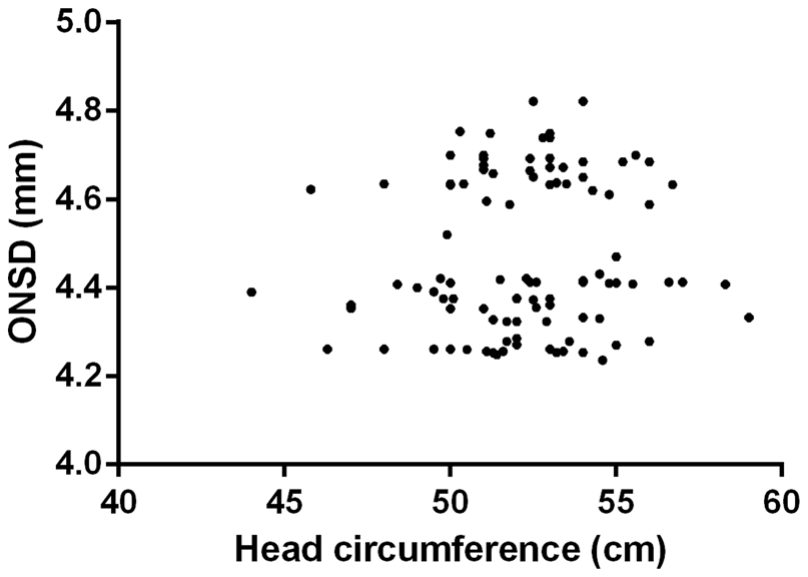
Optic nerve sheath diameter (ONSD) and head circumference.

There was no relationship between ONSD and age (R^2^ = 0.0093, p = 0.27, **figure 2**) or ONSD and head circumference (R^2^ = 0.011, p = 0.31, **figure 3**), and no difference in ONSD between males and females (p = 0.47). There were also no differences in individual's mean measurements taken in the horizontal or vertical planes (p = 0.99), or between left and right eyes (p = 0.12). The maximum difference between mean measurements in horizontal and vertical planes was 0.093 mm and between left and right eyes 0.13 mm.

The mean (95% CI) difference in individual ONSD measurements from the overall mean for each subject was 0.19 (0.17–0.20) mm. Repeating each measurement three times gave a mean difference of 0.07 (0.06–0.08) mm from the overall mean for each subject. The mean (95%) coefficient of variation of the multiple ONSD readings for each individual was 1.19 (1.09–1.29) % and the typical error of measurement 0.054 (0.049–0.59) mm.

## Discussion

This study indicates the cut-off for the upper limit of normal ONSD in Bangladeshi people ≥4 years old is 4.75 mm. The normal range of ONSD was independent of age, gender, ultrasound beam orientation, head circumference and observer.

The range of ONSD in this study (4.24–4.83 mm) was higher and narrower than has usually been found previously. Examples from previous studies include 2.5–4.1 mm in 50 UK adults[11], 2.1–4.3 mm in 102 UK children,[12] 2.5–4.1 mm in 31 African children,[13] 2.9–4.3 mm in 20 German adults[15] and 2.2–4.9 mm in 26 Greek adults.[7] A study in Iran found similar values to the present study with a mean of 4.6 mm in normal subjects.[16] The differences in normal range between studies may be due to differences between ethnicities, although it is not possible to exclude subtle differences in methodology as a contributing factor. Examples might include variation in the angle or positioning of the probe or differences in resolution. The precision of measurement increases with increasing power of the ultrasound probe used.[17] This study used a relatively powerful 15 MHz probe which may have partially accounted for the relatively narrow normal range found.

The distribution of ONSDs in this population was bimodal with a cut-off between the two groups of 4.5 mm. This could not be explained by differences between investigators, genders or ethnic origin and warrants further investigation. Possible explanations might include two genetically distinct subpopulations or differences in nutrition in childhood, for example malnutrition causing growth retardation and a smaller ONSD. A consequence of this is that in those with ONSD<4.5 mm, an increase in ICP could produce an ONSD within the normal distribution of the second peak between 4.5 and 4.75 mm.

This study had several limitations. A direct measure of ICP was not included thus it is not known how well ONSD above the derived normal range predicts ICP. Previous studies have shown a reliable linear relationship.[18] The present study did not include any volunteers under 4 years of age. Previously it has been shown that ONSD increases with age under 4 years and most within the first year of life.[2], [12] Previous studies have also suggested a much smaller increase in ONSD to the end of childhood, although this was not corroborated by the present study. Each measurement was made three times from the same video by the same observer. This observer was not blinded to the other results from the same video and this may have reduced the variability in these measurements due to observer bias. As the two investigators measuring ONSD did so in different patients, it was not possible to determine interobserver variability in this study. However, there was no difference in the median or range of observations by the two investigators and previous studies have shown inter-observer agreement to be high.[10], [11]

## Conclusions

Ultrasonographic measurements of ONSD in Bangladeshi healthy volunteers have a narrow bimodal distribution. ONSD is independent of age (≥4 years), gender and head circumference. ONSD>4.75 mm in this population should be considered abnormal.
