# Correction: Transorbital Sonographic Evaluation of Normal Optic Nerve Sheath Diameter in Healthy Volunteers in Bangladesh

**DOI:** 10.1371/annotation/4e2f88bd-e836-4e0c-a3e0-f6062331702b

**Published:** 2014-01-03

**Authors:** Rapeephan R. Maude, Md Amir Hossain, Mahtab Uddin Hassan, Sophie Osbourne, Katherine Langan, Abu Sayeed, Mohammed Rezaul Karim, Rasheda Samad, Shyamanga Borooah, Bal Dhillon, Nicholas P. J. Day, Arjen M. Dondorp, Richard J. Maude

The names of the fourth and fifth authors are incorrectly merged. Please view the correct list of authors, affiliations and Citation below:

Rapeephan R. Maude^1^*, Md Amir Hossain^3^, Mahtab Uddin Hassan^3^, Sophie Osbourne^1^, Katherine Langan^1^, Abu Sayeed^3^, Mohammed Rezaul Karim^3^, Rasheda Samad^3^, Shyamanga Borooah^4^,Bal Dhillon^4^, Nicholas P. J. Day^1,2^, Arjen M. Dondorp^1,2^, Richard J. Maude^1,2,4^

1 Mahidol-Oxford Tropical Medicine Research Unit, Rajthewi, Bangkok, Thailand

2 Centre for Tropical Medicine, Nuffield Department of Medicine, University of Oxford, Oxford, United Kingdom

3 Chittagong Medical College Hospital, Chittagong, Bangladesh

4 College of Medicine and Veterinary Medicine, University of Edinburgh,Edinburgh, United Kingdom

Maude RR, Amir Hossain M, Hassan MU, Osbourne S, Langan K, et al. (2013) Transorbital Sonographic Evaluation of Normal Optic Nerve Sheath Diameter in Healthy Volunteers in Bangladesh. PLoS ONE 8(12): e81013. doi:10.1371/journal.pone.0081013

